# Estimation of scabies prevalence using simplified criteria and mapping procedures in three Pacific and southeast Asian countries

**DOI:** 10.1186/s12889-021-12039-2

**Published:** 2021-11-10

**Authors:** Shu Ki Tsoi, Susanna J. Lake, Li Jun Thean, Alexander Matthews, Oliver Sokana, Mike Kama, Salvador Amaral, Lucia Romani, Margot Whitfeld, Joshua R. Francis, Susana Vaz Nery, Michael Marks, John M. Kaldor, Andrew C. Steer, Daniel Engelman

**Affiliations:** 1 Tropical Diseases, Murdoch Children’s Research Institute, Melbourne, Australia; 2 Department of Paediatrics, University of Melbourne, Melbourne, Australia; 3 Melbourne Children’s Global Health, Royal Children’s Hospital, Melbourne, Australia; 4 Royal Darwin Hospital, Darwin, Australia; 5 Ministry of Health and Medical Services, Honiara, Solomon Islands; 6 Ministry of Health, Dinem House, Suva, Republic of Fiji; 7 Menzies School of Health Research, Charles Darwin University, Darwin, Australia; 8 The Kirby Institute, University of New South Wales, Sydney, Australia; 9 St Vincent’s Hospital, University of New South Wales, Sydney, Australia; 10 Department of Paediatrics, Royal Darwin Hospital, Darwin, Australia; 11 Clinical Research Department, Faculty of Infectious and Tropical Diseases, London School of Hygiene & Tropical Medicine, London, UK; 12 Hospital for Tropical Diseases, University College London Hospital, London, UK

**Keywords:** Scabies, Diagnosis, Mapping, Neglected tropical diseases, Epidemiological monitoring, Cross-sectional studies

## Abstract

**Background:**

Scabies causes considerable morbidity in disadvantaged populations. The International Alliance for the Control of Scabies (IACS) published consensus criteria in 2020 to standardize scabies diagnosis. However, these criteria are complex, and a WHO informal consultation proposed simplified criteria for mapping, to identify regions of high prevalence as targets for mass drug administration. We aimed to investigate the accuracy of simplified criteria in determining scabies prevalence, compared to the 2020 IACS criteria.

**Methods:**

We obtained data relating to demographics, relevant history and skin lesions from all-age prevalence surveys from Fiji (*n* = 3365) and Solomon Islands (*n* = 5239), as well as school-aged children in Timor-Leste (*n* = 1043). We calculated prevalence using the 2020 IACS criteria and simplified criteria and compared these disease estimates.

**Results:**

There was no significant difference in the pooled prevalence using the two methods (2020 IACS criteria: 16.6%; simplified criteria: 15.6%; difference = 0.9, [95% CI -0.1, 2.0]). In Timor-Leste, the prevalence using simplified criteria was lower (26.5% vs 33.8%). Simplified criteria had a sensitivity of 82.3% (95% CI 80.2, 84.2) and specificity of 97.6% (95% CI 97.2, 97.9) compared to the 2020 IACS criteria.

**Conclusions:**

The scabies prevalence estimation using simplified criteria was similar to using the 2020 IACS criteria in high prevalence, tropical countries. The prevalence estimation was lower in the school-based survey in Timor-Leste. Mapping using simplified criteria may be a feasible and effective public health tool to identify priority regions for scabies control. Further work assessing use of simplified criteria for mapping in a field setting should be conducted.

**Supplementary Information:**

The online version contains supplementary material available at 10.1186/s12889-021-12039-2.

## Background

Scabies is a skin condition caused by the microscopic ectoparasite *Sarcoptes scabiei* var. *hominis*. It affects an estimated 150–200 million people worldwide [1], with an estimated annual incidence globally in 2019 of 565 (499–634) million cases and disease burden of 4.84 million disability-adjusted life-years [2]. Infestation with the scabies mite increases the risk of secondary bacterial complications such as impetigo, complicated skin and invasive infections, and immune-mediated conditions such as post-streptococcal glomerulonephritis and acute rheumatic fever [3]. Scabies is classified as a Neglected Tropical Disease (NTD) by the World Health Organization (WHO) warranting large-scale action for public health control [4–6]. Ivermectin-based mass drug administration (MDA) is effective for disease control in communities with high scabies prevalence (≥10%) [7–9]. Thus, a priority in the strategy for scabies control is establishing standardised diagnostic and survey methods to identify target areas for MDA.

While scabies is definitively diagnosed with direct microscopic visualisation of the mite or its products from skin scrapings, this test is not suitable in field settings due to cost, requirements for training and access to equipment, and low sensitivity [10, 11]. To standardise diagnosis, the International Alliance for the Control of Scabies (IACS) developed consensus criteria (2020 IACS criteria, Table S1) encompassing varying levels of diagnostic complexity for use in a variety of research, clinical or public health settings [12, 13]. For public health settings where direct visualisation techniques are not possible, a diagnosis of ‘Clinical Scabies’ or ‘Suspected Scabies’ using the 2020 IACS criteria requires the examining field worker to take a brief history of itch and contacts and do a skin examination. Initial validation of clinical assessment for non-expert examiners using the 2020 IACS criteria has shown moderate sensitivity and good specificity [14, 15].

However, assessment of contact history can be time consuming, and may not be reliable [15]. Simplified criteria defining ‘typical lesions in a typical distribution, with or without itch’ as scabies cases, were proposed by the 2019 WHO Informal Consultation on a Framework for Scabies Control as an alternative, based on the assumption that these criteria would be more efficient and feasible for rapid mapping purposes as they utilised an abbreviated examination and omitted assessment of contact history [16]. However, the accuracy of prevalence estimates using these simplified criteria is not known, and an evaluation was identified as an operational research priority by the WHO Informal Consultation [16, 17]. A high scabies prevalence has previously been identified in several Pacific and southeast Asian countries, including Fiji, Solomon Islands and Timor-Leste [5]. We aimed to evaluate the accuracy of prevalence estimations obtained by simplified criteria compared to the 2020 IACS criteria in surveys completed in these countries. Further, we aimed to investigate whether these estimates differ by country, sex, or age. Lastly, we aimed to explore the accuracy of alternatives to the proposed simplified criteria.

## Methods

### Setting

Fiji and Solomon Islands are Pacific Island countries with estimated populations in 2019 of 890,000 and 670,000 respectively [18], Timor-Leste is a southeast Asian country composed of the eastern half of the island of Timor, Atauro Island, Jaco Island and the enclave of Ambeno, and had an estimated population of 1.3 million in 2019 [18]. A national survey of scabies and impetigo in Fiji in 2017 estimated scabies prevalence of 18.5% [19]. Although no nationwide data are available for Solomon Islands and Timor-Leste, previous regional surveys between 2014 and 2016 estimate prevalence of 18.2–19.2%, and 17.0%, respectively [8, 20, 21].

### Study population and procedures

In this study of diagnostic accuracy [22], we utilised data collected during three prevalence surveys for scabies from Fiji, Solomon Islands and Timor-Leste [23–25]. De-identified data were collated from the three countries into a single dataset, including details on age, sex, country and findings of skin examination and history.

The three surveys were designed and conducted using identical methods for data collection and diagnosis. Survey procedures and sample size calculations are reported in detail elsewhere [23–26]. In brief, the studies in Solomon Islands and Fiji were community prevalence surveys involving participants of all ages. In Solomon Islands, residents from 20 villages in the Western Province were surveyed between May and July 2019, resulting in a total of 5239 participants. In Fiji, 3365 participants from 16 communities in the Northern Division were recruited in May 2019 [24]. In Timor-Leste, a school-based survey was conducted between April and May 2019, and enrolled a total of 1043 school children and their siblings aged less than 19 years of age from schools across three different municipalities [23].

A standardised training program was used to train nurses and doctors across the three sites, based on an established program [15]. In brief, local healthcare workers completed a one-week training program incorporating interactive tutorials on the features of scabies, based on the clinical categories of the 2020 IACS criteria which are the recommended standards for prevalence surveys [12]. This was followed by a written assessment of clinical photographs, requiring a minimum pass rate of 80%, and supervised practical sessions [23]. Demographic data relating to sex, age, locality and ethnicity were recorded. Positive contact history was elicited by asking participants whether they had household or other close contacts with itch, or with a typical scabies rash. Participants were shown photos of typical scabies rashes for reference. The skin of the head and neck, and upper and lower limbs was then inspected. The groin, buttocks, axillae and torso were not routinely examined. Examination of the trunk was included for children aged less than two years, consistent with the typical distribution of scabies in this group [12]. Examiners recorded the number of typical or atypical scabies lesions present and their location. Dermoscopy, skin scrapings and microscopy were not used as they are not usually available in clinical practice in these settings.

### Case definitions

The reference standard for diagnosis was the clinical categories of the 2020 IACS criteria (Table 1) [12]. Cases were classified as positive for scabies if they fulfilled either the criteria for ‘Clinical Scabies’ (subcategory B3), or ‘Suspected Scabies’ (subcategories C1 or C2), based on lesion appearance, lesion distribution, history of itch and contact history. Categories of the 2020 IACS criteria that require examination of the groin or specialised equipment and training, were not included (Table S1).

**Table 1:** Scabies diagnostic criteria used in study

**1. Reference Standard: 2020 IACS criteria (clinical categories used in study)**	
Subcategory B3: Typical lesions in a typical distribution and two history features	
Subcategory C1: Typical lesions in a typical distribution and one history features	
Subcategory C2: Atypical lesions or atypical distribution and two history features	
*History features: itch; positive contact history	
**2. Index Tests**	
A. **Simplified criteria** Typical lesions in a typical distribution, with or without itch	
B. **Alternative simplified criteria 1 (ASC1)** At least one of: i. Typical lesions in a typical distribution, with or without itch ii. Atypical lesions in a typical distribution, with itch	
C. **Alternative simplified criteria 2 (ASC2)** At least one of: i. Typical lesions in a typical distribution, with or without itch ii. Atypical lesions in a typical distribution, with or without itch	
D. **Alternative simplified criteria 3 (ASC3)** Itch	
E. **Alternative simplified criteria 4 (ASC4)** Itch and positive contact history	

Diagnosis using the simplified criteria was considered the index test. Simplified criteria were taken from the WHO Informal Consultation recommendations as ‘typical lesions and distribution of scabies on exposed skin, with or without itch’ [16]. The simplified criteria differs from the 2020 IACS criteria in that contact history and atypical lesions are not included (Table 2). Training requirements for examiners for simplified criteria may thus be reduced.

**Table 2:** Comparison of simplified diagnostic criteria to 2020 IACS criteria

	2020 IACS criteria	Simplified criteria
**History Features**		
**Itch**	Included. At least one of Itch or Contact History required	Included; not required
** Contact History**	Included. At least one of Itch or Contact History required	Not included
**Skin Examination**		
**Body Regions**	Whole body, where feasible Limited examination of exposed areas may be appropriate in field surveys	Frequently exposed areas only
**Lesion Appearance**	Atypical scabies lesions can be included if there is a typical distribution and both history features are present	Typical scabies lesions required
**Lesion Distribution**	Atypical distribution can be included where there are typical scabies lesions and both history features are present	Typical scabies distribution required
**Implementation Features**		
**Specialised Equipment**	Direct visualisation equipment for confirmed scabies, where feasible	No
**Examiner Minimum Level**	Experienced healthcare worker (nurse or physician)	Community health worker
**Training**	Longer, more specialised training and accreditation Specialised training for direct visualisation (microscopy or dermoscopy)	Brief training package
**Examples of Suitable Settings**	Clinical practice Clinical trials Accurate determination of prevalence (e.g., sentinel sites, impact surveys)	Community survey/mapping Defining areas where prevalence is likely to be ≥ 10% and a mass drug administration strategy may be recommended

Four alternative index tests were also investigated (Table 1). Two tests were chosen in an effort to capture cases which were classified as ‘Suspected Scabies’ by the 2020 IACS criteria, whilst remaining practical to implement in the field setting. These test criteria included ‘Atypical lesions in a typical distribution of scabies in frequently exposed skin areas, with itch (ASC1)’, and ‘Atypical lesions in a typical distribution of scabies in frequently exposed skin areas, with or without itch (ASC2)’. Two further alternatives, which would not require any skin inspection were also explored (ASC3: ‘Presence of itch’ and ASC4: ‘Presence of itch and positive contact history’). Based on each study participant’s recorded data, they were classified as either having or not having scabies, against each of the 2020 IACS criteria, simplified criteria, and the four alternative simplified criteria.

### Statistical analysis

Participants were excluded from analysis if data were missing (Fig. S1). We used descriptive statistics to calculate the prevalence of scabies overall, as well as for each country, sex and age group. We categorised age groups to enable comparison of children aged less than two years and children aged two to four years, as these can have a different clinical pattern of scabies [12]. Older ages were divided to give approximately even numbers of participants in each group. The difference in prevalence estimates was calculated, with 95% confidence intervals. We compared sex and age groups using relative risk [27]. Diagnostic accuracy of the simplified criteria strategy was analysed by calculating sensitivity and specificity compared with the 2020 IACS criteria, with 95% confidence intervals. Cohen’s kappa (κ) statistic was used to evaluate the inter-rater agreement for the classification of scabies using the different criteria. In further secondary analysis, we evaluated the four alternative simplified criteria by calculating prevalence estimates and sensitivity and specificity for each set of criteria. We used Stata/IC 16.1 (Statacorp LP, College Station Tx, USA) for all analysis.

## Results

A total of 9632 participants were recruited from the three studies. Following exclusion of 120 participants with incomplete data, a total of 9526 participants were included in the analysis (Fig. S1). The majority of participants (54.8%) were from the Solomon Islands, with fewer from Fiji (35.2%) and Timor-Leste (10.0%). There were slightly fewer males included (47.6%). The median age of participants was 15 years old (interquartile range 8–38 years old, range 0–98 years old) and the largest proportion of participants ranged from 5 to 14 years old (35.1%, Table 3). There was a higher proportion of participants aged less than 15 years old in the Solomon Islands survey compared to the 2009 national census (48.0% vs 41.0%) [28]. The age distribution of participants from Timor-Leste was different (median 10 years old; interquartile range 8–11 years old) as that survey only included children. There were no substantial differences in age or sex structure of the sample population in Fiji compared to the overall population reported in the 2017 census.

**Table 3:** Demographics of participants

	Solomon Islands	Fiji	Timor-Leste	Total
**Sex**				
Male	2473 (47.3%)	1625 (48.5%)	439 (46.2%)	4537 (47.6%)
Female	2751 (52.7%)	1726 (51.5%)	512 (53.8%)	4989 (52.4%)
**Age (years)**				
0–1	263 (5.0%)	171 (5.1%)	4 (0.4%)	438 (4.6%)
2–4	541 (10.4%)	258 (7.7%)	19 (2.0%)	818 (8.6%)
5–9	900 (17.2%)	403 (12.0%)	438 (46.1%)	1741 (18.3%)
10–14	804 (15.4%)	310 (9.3%)	482 (50.7%)	1596 (16.8%)
15–29	990 (19.0%)	719 (21.5%)	8 (0.8%)	1717 (18.0%)
30–49	1051 (20.1%)	809 (24.1%)	0	1860 (19.5%)
≥ 50	675 (12.9%)	681 (20.3%)	0	1356 (14.2%)
**Total**	5224 (54.8%)	3351 (35.2%)	951 (10.0%)	9526 (100.0%)

### Prevalence by 2020 IACS criteria

Using the 2020 IACS criteria, 1577 individuals (16.6%, [95% CI 15.8, 17.3], Table 4) were classified as cases of scabies. This included 880 cases (55.8% of cases) of ‘Clinical Scabies’, and 697 cases (44.2% of cases) of ‘Suspected Scabies’ (C1: 418 cases, 26.5% of cases; and C2: 279 cases, 17.7% of cases, Table 5). The prevalence of ‘Suspected Scabies subcategory C2’ in Timor-Leste was 8.9% (26.5% of cases), 2.7% in Solomon Islands (18.2% of cases) and 1.5% in Fiji (10.9% of cases, Table 5). Itch was present in 1470 cases (93.2% of cases), and contact history in 1266 (80.3% of cases, Table 5) [23].

**Table 4:** Comparison of prevalence estimates between 2020 IACS criteria and simplified criteria

	2020 IACS criteria		Simplified criteria		Difference between prevalence estimates (95% CI)	Kappa coefficient (κ, 95% CI)
	Scabies n	Prevalence % (95% CI)	Scabies n	Prevalence % (95% CI)		
**Total** (*n* = 9526)	1577	16.6 (15.8, 17.3)	1490	15.6 (14.9, 16.4)	0.9 (−0.1, 2.0)	0.82 (0.80–0.83)
**Country**						
Solomon Islands (*n* = 5224)	786	15.0 (14.1, 16.0)	788	15.1 (14.1, 16.1)	−0.0 (−1.4, 1.3)	0.79 (0.77–0.80)
Fiji (*n* = 3351)	470	14.0 (12.9, 15.2)	450	13.4 (12.3, 14.6)	0.6 (−1.15, 2.2)	0.90 (0.88–0.91)
Timor-Leste (*n* = 951)	321	33.8 (30.8, 36.8)	252	26.5 (23.8, 29.4)	7.3 (3.1, 11.4)	0.75 (0.73–0.77)
**Sex**						
Male (*n* = 4537)	804	17.7 (16.6, 18.9)	772	17.0 (15.9, 18.1)	0.7 (−0.9, 2.3)	0.82 (0.81–0.84)
Female (*n* = 4989)	773	15.5 (14.5, 16.5)	718	14.4 (13.4, 15.4)	1.1 (−0.3, 2.5)	0.81 (0.80–0.83)
**Age (years)**						
0–1 (*n* = 438)	124	28.3 (24.3, 32.7)	135	30.8 (26.7, 35.3)	−2.5 (−8.6, 3.5)	0.84 (0.83–0.86)
2–4 (*n* = 818)	212	25.9 (23.0, 29.0)	220	26.9 (24.0, 30.0)	−1.0 (−5.3, 3.3)	0.81 (0.80–0.83)
5–9 (*n* = 1741)	450	25.8 (23.8, 28.0)	440	25.3 (23.3, 27.4)	0.6 (−2.3, 3.5)	0.81 (0.79–0.82)
10–14 (*n* = 1596)	336	21.1 (19.1, 23.1)	319	20.0 (18.1, 22.0)	1.1 (−1.7, 3.9)	0.78 (0.77–0.80)
15–29 (*n* = 1717)	180	10.5 (9.1, 12.0)	163	9.5 (8.2, 11.0)	1.0 (−1.0, 3.0)	0.80 (0.79–0.82)
30–49 (*n* = 1860)	158	8.5 (7.3, 9.9)	123	6.6 (5.6, 7.8)	1.9 (0.2, 3.6)	0.83 (0.81–0.84)
≥ 50 (*n* = 1356)	117	8.6 (7.2, 10.2)	90	6.6 (5.4, 8.1)	2.0 (−0.0, 4.0)	0.82 (0.80–0.83)

CI = confidence interval

**Table 5:** Breakdown of scabies cases by 2020 IACS Criteria

	History Features		Examination Features		2020 IACS Criteria			
					Total Scabies	Subcategory		
	Itch	Positive Contact History	Typical Lesions	Typical Distribution		B3	C1	C2
	n (%)	n (%)	n (%)	n (%)	n	n (%)	n (%)	n (%)
**Country**								
Solomon Islands	706 (89.8%)	662 (84.2%)	644 (81.9%)	785 (99.9%)	786	439 (55.9%)	204 (26.0%)	143 (18.2%)
Fiji	454 (96.6%)	325 (69.1%)	423 (90.0%)	466 (99.1%)	470	258 (54.9%)	161 (34.3%)	51 (10.9%)
Timor-Leste	310 (96.6%)	279 (86.9%)	236 (73.5%)	321 (100.0%)	321	183 (55.8%)	53 (16.5%)	85 (26.5%)
**Sex**								
Male	745 (92.7%)	645 (80.2%)	674 (83.8%)	802 (99.8%)	804	454 (56.5%)	218 (27.1%)	132 (16.4%)
Female	725 (93.8%)	621 (80.3%)	629 (81.4%)	770 (99.6%)	773	426 (55.1%)	200 (25.9%)	147 (19.0%)
**Age (years)**								
0–1	110 (88.7%)	94 (75.8%)	115 (92.7%)	124 (100.0%)	124	71 (57.3%)	44 (35.5%)	9 (7.3%)
2–4	205 (96.7%)	164 (77.4%)	186 (87.7%)	212 (100.0%)	212	131 (61.8%)	55 (25.9%)	26 (12.3%)
5–9	423 (94.0%)	369 (82.0%)	381 (84.7%)	450 (100.0%)	450	273 (60.7%)	108 (24.0%)	69 (15.3%)
10–14	307 (91.4%)	281 (83.6%)	271 (80.7%)	336 (100.0%)	336	187 (55.7%)	84 (25.0%)	65 (19.3%)
15–29	167 (92.8%)	134 (74.4%)	142 (78.9%)	179 (99.4%)	180	82 (45.6%)	59 (32.8%)	39 (21.7%)
30–49	146 (92.4%)	137 (86.7%)	121 (76.6%)	155 (98.1%)	158	85 (53.8%)	33 (20.9%)	40 (25.3%)
≥ 50	112 (95.7%)	87 (74.4%)	87 (74.4%)	116 (99.1%)	117	51 (43.6%)	35 (29.9%)	31 (26.5%)
**Total**	1470 (93.2%)	1266 (80.3%)	1303 (82.6%)	1572 (99.7%)	1577	880 (55.8%)	418 (26.5%)	279 (17.7%)

The prevalence of scabies (either ‘Clinical Scabies’ or ‘Suspected Scabies’) was 33.8% in Timor-Leste, 15% in Solomon Islands, and 14% in Fiji (Table 4). A higher proportion of males (17.7%) were classified as having scabies compared to females (15.5%, relative risk (RR) 1.1, [95% CI 1.0, 1.3]). Scabies was more commonly diagnosed in younger age groups, with a prevalence of 24.4% in children aged less than 15 years, compared to those aged 15 years or older (prevalence 9.2%; RR 2.6, [95% CI 2.4, 2.9]).

### Prevalence by simplified criteria

The differences in the prevalence estimates obtained by the 2020 IACS criteria and the simplified criteria were not significant overall (16.6% vs 15.6%, difference 0.9, [95% CI -0.1, 2.0], Table 4) or in Solomon Islands or Fiji. In Timor-Leste, the 2020 IACS criteria prevalence was higher than the simplified criteria prevalence (33.8% vs 26.5%, difference 7.3, [95% CI 3.1, 11.4], Table 4). There was very strong agreement between the two prevalence estimates overall (κ = 0.82, [95% CI 0.80, 0.83], Table 4). In Timor-Leste the agreement was moderately strong (κ = 0.75, [95% CI 0.73, 0.77]). There were no statistical differences between the estimates using the two methods by sex or age groups.

### Sensitivity and specificity of simplified criteria

Overall, simplified criteria had a sensitivity of 82.3% (95% CI 80.2, 84.2) and specificity of 97.6% (95% CI 97.2, 97.9, Table 6) compared to the 2020 IACS criteria. There were 279 false negative cases (2.9% of all participants), all of whom had itch and a positive contact history but atypical features in either lesion appearance or distribution (2020 IACS criteria subcategory C2), thus failing to meet the simplified criteria definition. As contact history is not included in the simplified criteria, these cases were categorised as negative. The 192 false positives (2.0% of all participants) had typical lesions in a typical distribution but neither itch nor contact history (at least one of which is required by the 2020 IACS criteria).

**Table 6:** Diagnostic accuracy of simplified diagnostic compared to 2020 IACS criteria

	TP	TN	FP	FN	Sn % (95% CI)	Sp % (95% CI)
**Country**						
Solomon Islands (*n* = 5224)	643	4293	145	143	81.8 (78.9, 84.4)	96.7 (96.2, 97.2)
Fiji (*n* = 3351)	419	2850	31	51	89.1 (86.0, 91.8)	98.9 (98.5, 99.3)
Timor-Leste (*n* = 951)	236	614	16	85	73.5 (68.3, 78.3)	97.5 (95.9, 98.5)
**Sex**						
Male (*n* = 4537)	672	3633	100	132	83.6 (80.8, 86.1)	97.3 (96.7, 97.8)
Female (*n* = 4989)	626	4124	92	147	81.0 (78.0, 83.7)	97.8 (97.3, 98.2)
**Age (years)**						
0–1 (*n* = 438)	115	294	20	9	92.7 (86.7, 96.6)	93.6 (90.3, 96.1)
2–4 (*n* = 818)	186	572	34	26	87.7 (82.5, 91.8)	94.4 (92.2, 96.1)
5–9 (*n* = 1741)	381	1232	59	69	84.7 (81.0, 87.9)	95.4 (94.1, 96.5)
10–14 (*n* = 1596)	271	1212	48	65	80.7 (76.0, 84.7)	96.2 (95.0, 97.2)
15–29 (*n* = 1717)	141	1515	22	39	78.3 (71.6, 84.1)	98.6 (97.8, 99.1)
30–49 (*n* = 1860)	118	1697	5	40	74.7 (67.2, 81.3)	99.7 (99.3, 99.9)
≥ 50 (*n* = 1356)	86	1235	4	31	73.5 (64.5, 81.2)	99.7 (99.2, 99.9)
**Total** (*n* = 9526)	1298	7757	192	279	82.3 (80.2, 84.2)	97.6 (97.2, 97.9)

TP = true positive, TN = true negative, FP = false positive, FN = false negative, Sn = sensitivity, Sp = specificity, CI = confidence interval

Sensitivity and specificity did not vary between males and females. The sensitivity was highest in children aged less than two years (92.7%) and decreased with age (73.5% in those ≥50 years). Specificity increased with age (93.6% in children aged less than two years to 99.7% in those aged ≥30 years, Table 6).

### Alternative simplified criteria

The proportion of individuals classified as having scabies estimated by alternative simplified criteria ASC1, ASC2 and ASC3 were significantly higher than estimates using 2020 IACS criteria (ASC1: 20.3%, difference = − 3.7% [95% CI −4.8, − 2.6], ASC2: 23.8%, difference = − 7.2% [95% CI −8.3, − 6.1], ASC3: 27.8%, difference = − 11.2% [95% CI −12.4, − 10.0], Table S2). ASC4 produced the most similar prevalence estimate overall of 16.7% (difference = − 0.1%, [95% CI −1.2, 0.9]) however greatly overestimated scabies prevalence in those younger than five years old (difference = 6.1%, [95% CI 2.7, 9.4]). Use of the alternate criteria led to an increase in sensitivity for ASC1 (99.7%, [95% CI 99.3, 99.9]), ASC2 (99.7%, [95% CI 99.3, 99.9]) and ASC3 (93.2%, [95% CI 91.9, 94.4]), however all four alternative criteria had lower specificity (Table S3).

## Discussion

Our results suggest that the simplified criteria proposed by the WHO Informal Consultation can be used to accurately estimate the prevalence of scabies during rapid mapping and surveys, with a similar prevalence estimate when compared to assessment using the 2020 IACS criteria. The simplified criteria had a sensitivity of 82% and specificity of 98%. We believe this accuracy is acceptable for the purposes of surveys, where public health decisions are made based on community prevalence, rather than treatment decisions based on individual diagnoses.

Rapid mapping aims to identify areas where the community prevalence of scabies is ≥10% (or ≥ 15% in school-aged children, if a school-survey method is used) for implementation of MDA programs [16]. Based on our data in these three countries where scabies prevalence was > 10%, use of simplified criteria as opposed to 2020 IACS criteria would not lead to a different recommendation for where MDA should be implemented. This study demonstrates that the simplified criteria may be useful in regions with a high scabies prevalence. This is an important finding as these are the regions where the public health control of scabies using MDA is being prioritised, but where there are currently very little or no estimates of prevalence. However, it is possible that in populations where scabies prevalence is close to the recommended MDA starting threshold, choice of criteria may impact public health decision making.

Accurate mapping strategies are important to identify and prioritise areas for scabies control and MDA, but these methods need to be feasible at large scale. Rapid mapping strategies, using simplified criteria, have been successfully used for the control of other NTDs including trachoma, schistosomiasis, lymphatic filariasis and onchocerciasis [29, 30]. Similarly, in 1994, a UK working party developed simplified diagnostic criteria for atopic dermatitis to be used in epidemiological studies [31–34]. The simplified criteria for scabies comprise a brief examination of frequently exposed skin, which may facilitate implementation at large scale with limited resources by using less specialised local healthcare workers and reducing the number of interpreters required. Studies comparing the accuracy of examination of exposed areas of the skin to examination of the whole body found that examination of exposed areas had close to 90% sensitivity [14, 35]. Omitting contact history and consideration of atypical lesions should further simplify training and assessment in the field. In addition, it may increase recruitment rates by omitting examination of sensitive body areas and reducing the time burden placed on participants. Our exploration of alternatives to the simplified criteria showed that it is possible to improve sensitivity by including cases with atypical lesions. However, three of the four of these alternative methods (ASC1, ASC2, ASC3) overestimated the prevalence of scabies. Therefore, our data support the current recommendations for the simplified criteria. Prevalence estimates using ASC4 (presence of itch and contact history) were similar to those using 2020 IACS criteria. However, itch is a highly non-specific symptom which is caused by many common conditions, and is variably reported, including in young children. Further studies would be required to investigate whether an assessment method that omits examination of the skin could be a feasible alternative to the proposed simplified criteria.

The simple, brief nature of scabies assessment may facilitate integration with surveys for other NTDs and other health programmes [36, 37]. Epidemiological mapping of onchocerciasis (REMO) integrates geographical and environmental risk factors with mapping data to establish zones of endemicity [38]. Although no such risk factors have been established for scabies, and scabies is not a vector-borne disease, this could be explored through future research.

Our study has limitations. First, the three surveys we included are from tropical, island countries with a very high scabies prevalence. Our results may not be generalisable to other settings such as temperate climates, mainland populations, highly urbanised environments or to lower-prevalence settings. Second, the Timor-Leste survey only enrolled school-aged children and their siblings. Scabies is more prevalent in children, which may have contributed to the difference in results between surveys. There is also a high prevalence of secondary impetiginized scabies in this population [19, 39, 40]. These cases may have lesions of atypical appearance or distribution, which are not classified as scabies using the simplified criteria. Similarly, scabies can present in more atypical forms in the elderly population, which may have contributed to the lower sensitivity seen in those aged 50 years and older [41]. Third, the current recommended threshold to cease MDA is a prevalence of < 2% [16], and further evidence is needed to compare the accuracy of simplified criteria in lower-prevalence settings. Fourth, we were only able to compare simplified criteria with the ‘Clinical and Suspected Scabies’ levels of the 2020 IACS criteria, as the ‘Confirmed Scabies’ level, which requires direct mite visualisation was not feasible to include in these surveys. The absence of an appropriate reference standard is a limitation for all studies of diagnostic accuracy for scabies, and further development of objective diagnostic tests that can be feasibly used in the field is required. Fifth, we compared the accuracy of the criteria based on an analysis of the components collected during a single assessment in the field, in which health workers were trained to assess participants according to 2020 IACS clinical criteria. It is possible that knowledge of contact history may have influenced the classification of skin lesions in some cases, and it is not known how a different training program and survey method, based on the simplified criteria, would affect the recording of scabies lesions. Finally, examination was conducted by healthcare workers with limited experience, and the sensitivity of scabies diagnosis may be lower, particularly for mild forms of scabies [15].

## Conclusions

There was no significant difference between the prevalence of scabies using simplified diagnostic criteria, as recommended in the informal WHO Framework, compared with the 2020 IACS criteria in the pooled survey results from these three tropical island populations. Implementation of simplified criteria may be an efficient and accurate way to facilitate rapid mapping to determine high prevalence areas for scabies control. Further work is needed to investigate the accuracy in urbanised and lower-prevalence populations, and to evaluate the implementation of simplified criteria in the field setting.

## Supplementary Information

**Additional file 1: Table S1.** Summary of the 2020 IACS consensus criteria for diagnosis of scabies [12]**.** List of 2020 IACS criteria and whether they were utilised in surveys.

**Additional file 2: Table S2.** Scabies prevalence estimates by alternative simplified criteria. Prevalence estimates of scabies cases by 2020 IACS criteria and alternative simplified criteria by country, sex and age.

**Additional file 3: Table S3.** Sensitivity and specificity of alternative simplified criteria. Sensitivity and specificity of alternative simplified criteria by country, sex and age.

**Additional file 4: Table S4**. STARD 2015 Checklist. Fulfilment of STARD criteria by study.

**Additional file 5: Fig. S1.** Study flow diagram outlining number of participants recruited in study by country.

## Data Availability

De-identified individual participant data that underlies the results reported in this study will be made available to researchers whose proposed use of the data has been approved. Proposals should be directed to Daniel.Engelman@rch.org.au to gain access.
