# Supplementary material for: Estimation of scabies prevalence using simplified criteria and mapping procedures in three Pacific and southeast Asian countries
Source: BMC Public Health. 2021 Nov 10;21:2060. doi: 10.1186/s12889-021-12039-2 (PMC8579609; doi:10.1186/s12889-021-12039-2)
Supplement: Supplementary file 1 — Additional file 1: Table S1. Summary of the 2020 IACS consensus criteria for diagnosis of scabies [12]. List of 2020 IACS criteria and whether they were utilised in surveys. [file 12889_2021_12039_MOESM1_ESM.docx]

**Table S1. Summary of the 2020 IACS consensus criteria for diagnosis of scabies [12]**

| **2020 IACS criteria** | **Used in survey** |
| --- | --- |
| **Category A: Confirmed scabies**  ***At least one of:*** |  |
| A1: Mites, eggs or faeces on light microscopy of skin samples | No |
| A2: Mites, eggs or faeces visualized on individual using high-powered imaging device | No |
| A3: Mite visualized on individual using dermoscopy | No |
| **Category B: Clinical scabies**  ***At least one of:*** |  |
| B1: Scabies burrows | No |
| B2: Typical lesions affective male genitalia | No |
| B3: Typical lesions in a typical distribution and two history features | Yes |
| **Category C: Suspected scabies**  One of: |  |
| C1: Typical lesions in a typical distribution and one history feature | Yes |
| C2: Atypical lesions or atypical distribution and two history features | Yes |
| **History features**  H1: Itch  H2: Positive contact history – at least one of:   1. Any contact with an individual diagnosed with crusted scabies 2. Close contact* with an individual diagnosed with scabies 3. Close contact* with an individual with itch that is not accounted for by another condition 4. Close contact* with an individual with typical scabies lesions in a typical distribution that are not accounted for by another condition | |

**Close contacts are defined as any of: 1. Individuals that sleep in the same dwelling. 2. Individuals that share a bed (including sexual partners).3. Children in the same classroom or who play closely together. 4. Adults with known skin-to-skin contact*
