# Supplementary material for: Estimation of scabies prevalence using simplified criteria and mapping procedures in three Pacific and southeast Asian countries
Source: BMC Public Health. 2021 Nov 10;21:2060. doi: 10.1186/s12889-021-12039-2 (PMC8579609; doi:10.1186/s12889-021-12039-2)
Supplement: Supplementary file 2 — Additional file 2: Table S2. Scabies prevalence estimates by alternative simplified criteria. Prevalence estimates of scabies cases by 2020 IACS criteria and alternative simplified criteria by country, sex and age. [file 12889_2021_12039_MOESM2_ESM.docx]

**Table S2. Scabies prevalence estimates by alternative simplified criteria**

|  | **2020 IACS** | **ASC1** | | **ASC2** | | **ASC3** | | **ASC4** | |
| --- | --- | --- | --- | --- | --- | --- | --- | --- | --- |
|  | **P %** | **P %** | **ΔP%** | **P %** | **ΔP%** | **P %** | **ΔP%** | **P %** | **ΔP%** |
|  | **(95% CI)** | **(95% CI)** | **(95% CI)** | **(95% CI)** | **(95% CI)** | **(95% CI)** | **(95% CI)** | **(95% CI)** | **(95% CI)** |
| **Total** (n=9526) | 16.6  (15.8, 17.3) | 20.3  (19.5, 1.1) | -3.7  (-4.8, -2.6) | 23.8  (22.9, 4.6) | -7.2  (-8.3, -6.1) | 27.8  (26.9,28.7) | -11.2  (-12.4, -10.0) | 16.7  (7.5, 16.0) | -0.1  (-1.2, 0.9) |
| **Country** |  |  |  |  |  |  |  |  |  |
| Solomon Islands (n=5224) | 15.0  (14.1, 16.0) | 19.6  (18.5, 20.7) | -4.5  (-6.0, -3.1) | 24.4  (23.3, 25.6) | -9.4  (-10.9, -7.7) | 23.9  (22.8, 25.1) | -8.9  (-10.4, -7.4) | 13.6  (12.5, 14.8) | -0.4  (-1.8, 0.9) |
| Fiji (n=3351) | 14.0  (12.9, 15.2) | 16.4  (15.2, 17.7) | -2.4  (-4.1, -0.7) | 17.3  (16.1, 18.7) | -3.3  (-5.1, -1.6) | 27.4  (25.9, 29.0) | -13.4  (-15.3, -11.5) | 15.5  (14.5, 16.5) | 0.4  (-1.2, 2.1) |
| Timor-Leste (n=951) | 33.8  (30.8, 36.8) | 37.9  (34.8, 41.0) | -4.1  (-8.4, 0.2) | 42.7  (39.6, 45.9) | -8.9  (-13.3, -4.6) | 50.1  (46.9, 53.2) | -16.3  (-20.7, -11.9) | 34.3  (31.3, 37.4) | -0.5  (-4.8, 3.7) |
| **Sex** |  |  |  |  |  |  |  |  |  |
| Male (n=4537) | 17.7  (16.6, 18.9) | 21.9  (20.7, 23.1) | -4.1  (-5.8, -2.5) | 25.3  (24.1, 26.6) | -7.6  (-9.3, -5.9) | 28.8  (27.5, 30.2) | -11.1  (-12.8, -9.4) | 17.3  (16.3, 18.5) | 0.4  (-1.2, 2.0) |
| Female (n=4989) | 15.5  (14.5, 16.5) | 18.8  (17.8, 20.0) | -3.3  (-4.8, -1.9) | 22.3  (21.2, 23.5) | -6.8  (-8.4, -5.3) | 26.8  (25.6, 28.0) | -11.3 (-12.9, -9.7) | 16.1  (15.1, 17.2) | -0.6  (-2.1, 0.8) |
| **Age (years)** |  |  |  |  |  |  |  |  |  |
| 0 – 1 (n=438) | 28.3  (24.3, 32.7) | 34.5  (30.2, 39.1) | -6.2  (-12.3, 0.0) | 39.7  (35.2, 44.4) | -11.4  (-17.6, -5.2) | 28.5  (24.5, 33.0) | -0.2  (-6.2, 5.7) | 19.2  (15.7, 23.1) | 9.1  (3.5, 14.7) |
| 2 – 4 (n=818) | 25.9  (23.0, 29.0) | 31.8  (28.7, 35.1) | -5.9  (-10.2, -1.5) | 36.3  (33.1, 39.7) | -10.4  (-14.8, -5.9) | 31.3  (28.2, 34.6) | -5.4  (-9.8, -1.0) | 21.5  (18.8, 24.5) | 4.4  (0.3, 8.5) |
| 5 – 9 (n=1741) | 25.8  (23.8, 28.0) | 31.6  (29.5, 33.9) | -5.8  (-8.8, -2.8) | 37.2  (35.0, 39.5) | -11.4  (-14.4, -8.3) | 35.7  (33.5, 38.0) | -9.8  (-12.9, -6.8) | 23.1  (21.2, 25.1) | 2.8  (-0.1, 5.6) |
| 10 – 14 (n=1596) | 21.1  (19.1, 23.1) | 26.1  (24.0, 28.3) | -5.1  (-8.0, -2.1) | 30.8  (28.5, 33.1) | -9.7  (-12.7, -6.7) | 30.8  (28.6, 33.1) | -9.8  (-12.8, -6.8) | 20.1  (18.2, 22.1) | 1.0  (-1.8, 3.8) |
| 15 – 29 (n=1717) | 10.5  (9.1, 12.0) | 13.0  (11.5, 14.7) | -2.5  (-4.7, -0.4) | 15.1  (13.5, 16.9) | -4.7  (-6.9, -2.4) | 21.3  (19.4, 23.3) | -10.8  (-13.3, -8.4) | 11.7  (10.3, 13.3) | -1.2  (-3.3, 0.9) |
| 30 – 49 (n=1860) | 8.5  (7.3, 9.9) | 9.5  (8.3, 10.9) | -1.0  (-2.9, 0.8) | 11.7  (10.3, 13.3) | -3.2  (-5.2, -1.3) | 21.8  (20.0, 23.8) | -13.3  (-15.6, -11.1) | 12.6  (11.2, 14.2) | -4.1  (-6.1, -2.2) |
| ≥50 (n=1356) | 8.6  (7.2, 10.2) | 11.3  (9.7, 13.1) | -2.7  (-4.9, -0.4) | 13.0  (11.3, 14.9) | -4.4  (-6.7, 2.0) | 27.9  (25.6, 30.4) | -19.3  (-22.1, -16.5) | 12.8  (11.1, 14.6) | -4.1  (-6.5, -1.8) |

ASC = alternative simplified criteria, P = prevalence, ΔP% = percentage difference in prevalence estimates, CI = confidence interval.
