# Supplementary material for: Estimation of scabies prevalence using simplified criteria and mapping procedures in three Pacific and southeast Asian countries
Source: BMC Public Health. 2021 Nov 10;21:2060. doi: 10.1186/s12889-021-12039-2 (PMC8579609; doi:10.1186/s12889-021-12039-2)
Supplement: Supplementary file 3 — Additional file 3: Table S3. Sensitivity and specificity of alternative simplified criteria. Sensitivity and specificity of alternative simplified criteria by country, sex and age. [file 12889_2021_12039_MOESM3_ESM.docx]

**Table S3. Sensitivity and specificity of alternative simplified criteria**

|  | **ASC1** | | **ASC2** | | **ASC3** | | **ASC4** | |
| --- | --- | --- | --- | --- | --- | --- | --- | --- |
|  | **Sn %** | **Sp %** | **Sn %** | **Sp %** | **Sn %** | **Sp %** | **Sn %** | **Sp %** |
|  | **(95% CI)** | **(95% CI)** | **(95% CI)** | **(95% CI)** | **(95% CI)** | **(95% CI)** | **(95% CI)** | **(95% CI)** |
| **Total** (n=9526) | 99.7  (99.3, 99.9) | 95.5  (95.0, 95.9) | 99.7  (99.3, 99.9) | 91.3  (90.7, 91.9) | 93.2  (91.9, 94.4) | 85.2  (84.4, 86.0) | 73.5  (71.2, 75.7) | 94.6  (94.0, 95.1) |
| **Country** |  |  |  |  |  |  |  |  |
| Solomon Islands (n=5224) | 99.9  (99.3, 100.0) | 94.7  (94.0, 95.3) | 99.9  (99.3, 100.0) | 88.9  (88.0, 89.8) | 89.8  (87.5, 91.8) | 87.7  (86.7, 88.7) | 74.0  (70.8, 77.1) | 94.9  (94.2, 95.5) |
| Fiji (n=3351) | 99.1  (97.8, 99.8) | 97.1  (96.4, 97.7) | 99.1  (97.8, 99.8) | 96.0  (95.2, 96.7) | 96.6  (94.5, 98.0) | 83.9  (82.5, 85.2) | 65.7  (61.3, 70.0) | 94.9  (94.0, 95.7) |
| Timor-Leste (n=951) | 100.0  (98.9, 100.0) | 93.8  (91.6, 95.6) | 100.0  (98.9, 100.0) | 86.5  (83.6, 89.1) | 96.6  (94.0, 98.3) | 73.7  (70.0, 77.1) | 83.5  (79.0, 87.4) | 90.8  (88.3, 92.9) |
| **Sex** |  |  |  |  |  |  |  |  |
| Male (n=4537) | 99.8  (99.1, 100.0) | 94.9  (94.2, 95.6) | 99.8  (99.1, 100.0) | 90.7  (89.7, 91.6) | 92.7  (90.6, 94.4) | 84.9  (83.7, 86.1) | 72.9  (69.7, 75.9) | 94.6  (93.9, 95.3) |
| Female (n=4989) | 99.6  (98.9, 99.9) | 96.0  (95.3, 96.5) | 99.6  (98.9, 99.9) | 91.8  (91.0, 92.6) | 93.8  (91.9, 95.4) | 85.5  (84.4, 86.5) | 74.1  (70.9, 77.2) | 94.5  (93.8, 95.2) |
| **Age (years)** |  |  |  |  |  |  |  |  |
| 0 – 1 (n=438) | 100.0  (97.1, 100.0) | 91.4  (87.7, 94.3) | 100.0  (97.1, 100.0) | 84.1  (79.6, 87.9) | 88.7  (81.8, 93.7) | 95.2  (92.2, 97.3) | 64.5  (55.4, 72.9) | 98.7  (96.8, 99.7) |
| 2 – 4 (n=818) | 100.0  (98.3, 100.0) | 92.1  (89.6, 94.1) | 100.0  (98.3, 100.0) | 86.0  (83.0, 88.6) | 96.7  (93.3, 98.7) | 91.6  (89.1, 93.7) | 74.1  (67.6, 79.8) | 96.9  (95.1, 98.1) |
| 5 – 9 (n=1741) | 100.0  (99.2, 100.0) | 92.2  (90.6, 93.6) | 100.0  (99.2, 100.0) | 84.7  (82.6, 86.6) | 94.0  (91.4, 96.0) | 84.7  (82.6, 86.6) | 76.0  (71.8, 79.9) | 95.4  (94.1, 96.4) |
| 10 – 14 (n=1596) | 100.0  (98.9, 100.0) | 93.6  (92.1, 94.9) | 100.0  (98.9, 100.0) | 87.7  (85.8, 89.5) | 91.4  (87.8, 94.1) | 85.3  (83.2, 87.2) | 75.0  (70.0, 79.5) | 94.6  (93.2, 95.8) |
| 15 – 29 (n=1717) | 99.4  (96.9, 100.0) | 97.1  (96.2, 97.9) | 99.4  (96.9, 100.0) | 94.7  (93.5, 95.8) | 92.8  (88.0, 96.1) | 87.1  (85.3, 88.7) | 67.2  (59.8, 74.0) | 94.8  (93.6, 95.9) |
| 30 – 49 (n=1860) | 98.1  (94.6, 99.6) | 98.7  (98.0, 99.2) | 98.1  (94.6, 99.6) | 96.3  (95.3, 97.1) | 92.4  (87.1, 96.0) | 84.7  (82.9, 86.4) | 79.1  (71.9, 85.2) | 93.5  (92.3, 94.7) |
| ≥50 (n=1356) | 99.1  (95.3, 100.0) | 97.0  (95.9, 97.9) | 99.1  (95.3, 100.0) | 95.2  (93.8, 96.3) | 95.7  (90.3, 98.6) | 78.5  (76.1, 80.7) | 70.1  (60.9, 78.2) | 92.7  (91.1, 94.0) |

ASC = alternative simplified criteria, Sn = sensitivity, Sp = specificity, CI = confidence interval.
