# Supplementary figures and images for: Estimation of scabies prevalence using simplified criteria and mapping procedures in three Pacific and southeast Asian countries
Source: BMC Public Health. 2021 Nov 10;21:2060. doi: 10.1186/s12889-021-12039-2 (PMC8579609; doi:10.1186/s12889-021-12039-2)

**Fig S1. Study flow diagram**


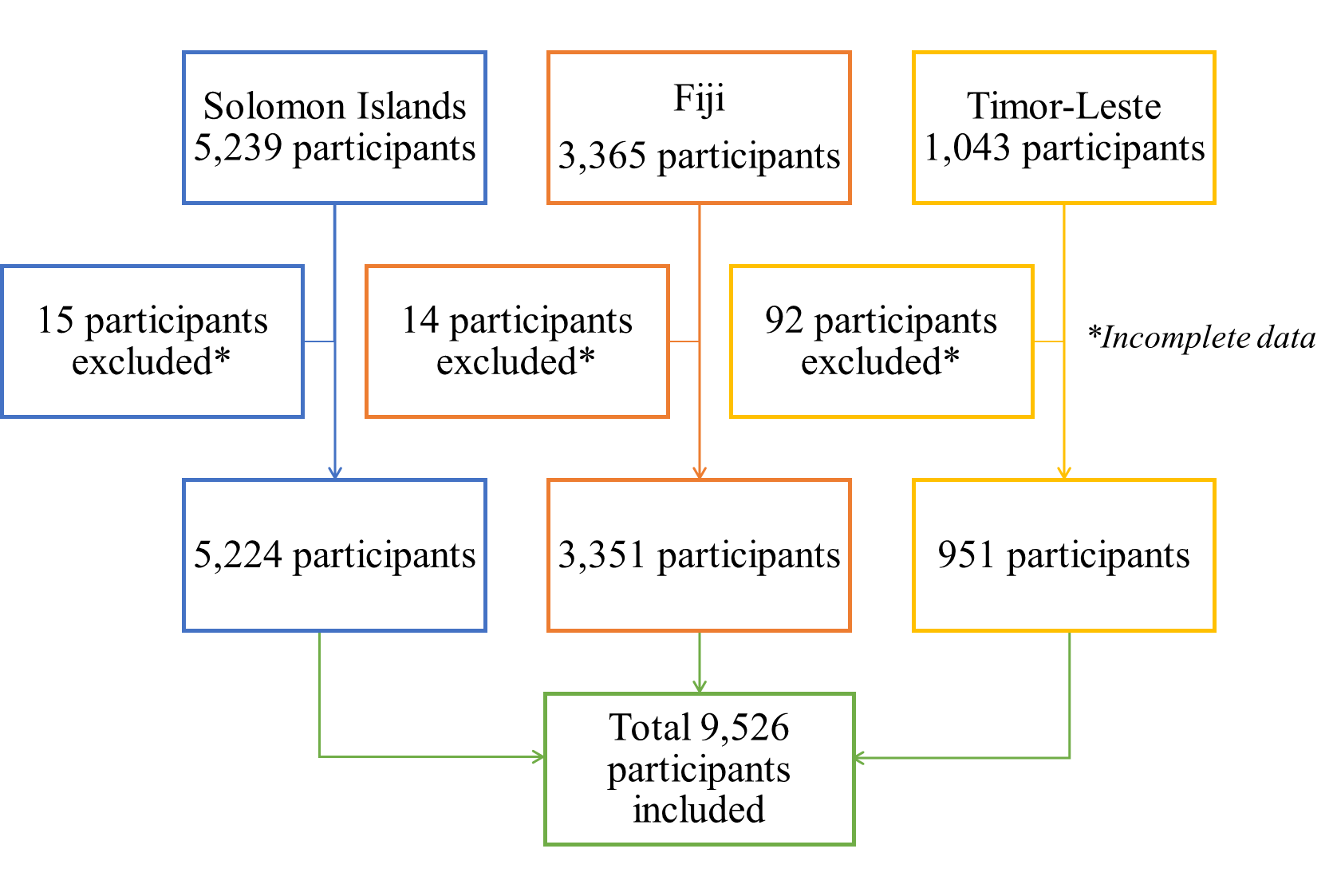

Supplement: Supplementary file 5 — Additional file 5: Fig. S1. Study flow diagram outlining number of participants recruited in study by country. [file 12889_2021_12039_MOESM5_ESM.docx]
